# Functional Cement-Based Composites Materials for Future Applications

**DOI:** 10.3390/ma18245610

**Published:** 2025-12-14

**Authors:** Jonathan Oti

**Affiliations:** School of Engineering, Faculty of Computing, Engineering and Science, University of South Wales, Pontypridd CF37 1DL, UK; jonathan.oti@southwales.ac.uk

## 1. Introduction

The global demand for cement-based materials continues to rise, particularly in emerging economies where infrastructure and housing development are accelerating. Cement remains one of the most widely used materials in the built environment, and its production is expected to grow despite increasing environmental regulations aimed at reducing carbon emissions [1,2,3,4,5]. Cement production alone contributes approximately 8% of global CO_2_ emissions, primarily due to the calcination of limestone and the energy-intensive nature of the process [6,7].

In response to these challenges, the construction industry is undergoing a transformation toward sustainability. This Special Issue brings together innovative and economically viable materials derived from alternative cement replacement strategies. It highlights interdisciplinary approaches to developing functional cement-based composites with reduced environmental impact. These include the use of waste plastic as coarse aggregate in geopolymer concrete, granite powder as partial cement replacement, and ground glass powder as a supplementary cementitious material.

Recent advancements have demonstrated the potential of geopolymer concrete (GPC) as a sustainable alternative to traditional Portland cement [8,9]. GPC offers superior mechanical properties, high thermal resistance, and up to 80% reduction in CO_2_ emissions [10]. Innovations such as nanomaterial integration, hybrid binder systems, and 3D printing are expanding its applications and performance [11]. Moreover, recycled cement technologies have shown promise in reducing emissions by up to around 61% while maintaining strength comparable to conventional cement [12].

These developments underscore the importance of continued research and collaboration across academia, industry, and policy. The papers in this Special Issue provide valuable insights for civil engineers, material scientists, and sustainability practitioners seeking advanced techniques and alternative approaches to low-carbon construction. By integrating performance-based assessments, life cycle analysis, and digital optimization tools, the industry can move toward net-zero emissions while maintaining the durability and versatility of cement-based materials [13]

## 2. An Overview of Published Articles

Huang et al. [14] presents an experimental study on dovetail profiled steel concrete composite shear walls (DPSCWs) connected using self-tapping screws, aiming to improve seismic resilience and construction efficiency. Traditional welded connections in thin-walled steel structures often suffer from deformation and are unsuitable for prefabrication. By introducing self-tapping screws, the study fills a gap in functional cement-based composites by offering a reliable, flexible, and damage-resistant connection method. The research evaluates hysteretic behavior, failure modes, ductility, and energy dissipation, and proposes a design method based on effective strip theory. The findings demonstrate that screw-connected DPSCWs meet seismic design standards, enhance deformation capacity, and simplify construction, contributing to the development of intelligent, resilient structural systems in civil engineering. The study concludes that self-tapping screw-connected DPSCWs show reliable seismic performance, meeting code requirements. Increasing screw quantity improves ductility and energy dissipation, while higher axial compression boosts stiffness but reduces deformability.

The article by Rogojsz and Rudnicki [15] explores the impact of various mineral additives, such as white and compacted microsilica, limestone flour, glass flour, basalt dust, and glass granulate, on the alkaline reactivity of aggregates in cement-based composites. The study addresses an important gap in civil engineering: the limited usability of reactive aggregates in concrete due to alkali–silica reactions (ASR), which compromise durability. By replacing 10% and 20% of cement with these additives, the authors demonstrate that certain materials, especially white microsilica, can significantly reduce ASR, enabling the use of lower-grade aggregates in infrastructure applications. This contributes to sustainable construction by expanding aggregate sources and reducing cement usage, aligning with environmental goals. The findings offer practical solutions for enhancing the performance and longevity of functional cement-based composites in road and bridge engineering. The work concludes that white microsilica significantly reduces aggregate reactivity, enabling use of reactive materials in road concrete. Other additives like glass flour and limestone also help. Compacted microsilica, however, increases reactivity and is unsuitable.

The third article is from Rudnicki [16], the study introduces a novel method for designing self-compacting concrete (SCC) with reduced cement content by optimizing aggregate packing. It addresses important gaps in functional cement-based composites for civil engineering by proposing a simplified, performance-driven design approach that minimizes voids and enhances durability. The study systematically evaluates 36 mix designs using the blocking method to achieve optimal aggregate proportions, reducing cement from 500 to 350 kg/m^3^ without compromising strength or freeze–thaw resistance. This contributes to sustainable construction by improving material efficiency, reducing environmental impact, and expanding SCC applicability in complex structural designs. The study concludes that optimizing aggregate packing in SCC design enables reduced cement use while maintaining strength and durability. The blocking method proves effective, offering sustainable, high-performance concrete with minimal voids and enhanced flow properties.

The fourth text published in this Special Issue is Zhang et al. [17], the study presents a novel approach to enhancing vegetation concrete, a cement-based composite used in ecological slope protection, by incorporating plant protein foaming agents. Traditional vegetation concrete suffers from consolidation, limiting root growth and nutrient uptake. The study fills this gap by introducing foam to improve porosity and reduce weight, while maintaining sufficient mechanical strength. Through physical, mechanical, and biological tests, the authors demonstrate that a foam volume of 20–30% optimizes plant growth and structural integrity. This innovation advances functional cement composites by balancing ecological performance with engineering requirements, offering a sustainable, lightweight, and porous solution for slope stabilization. It contributes to civil engineering by integrating environmental functionality into structural materials, addressing both ecological restoration and material efficiency. The study concludes that foamed vegetation concrete with 20–30% plant protein foam improves porosity, reduces weight, and supports plant growth while maintaining strength for slope protection. It offers a sustainable, efficient solution for ecological restoration.

Costafreda et al. [18] study, investigates a newly discovered natural zeolite-rich tuff in the Los Frailes Caldera, focusing on its mineralogical, chemical, and pozzolanic properties. Through advanced characterization techniques (XRD, SEM, XRF, TGA), the authors demonstrate that the material—rich in mordenite—exhibits high reactivity and mechanical strength when blended with Portland cement. The research addresses an important gap in sustainable civil engineering by offering a locally sourced, high-performance supplementary cementitious material (SCM) that enhances durability and reduces CO_2_ emissions. Unlike conventional SCMs, this zeolite-rich tuff shows superior purity and pozzolanicity, contributing to long-term strength gains in mortars and concretes. The findings support its use in functional cement-based composites, promoting eco-efficient construction and extending the life of existing mineral reserves. The main conclusions were that newly discovered zeolite-rich tuff shows excellent pozzolanic properties, high silica content, and strong mechanical performance, making it a viable supplementary cementitious material and promising for expanding Spain’s industrial reserves.

The work by Rudnicki and Stałowski [19] investigates on the performance of cement concrete pavements using low-emission cements (CEM II, III, V) to address environmental and durability challenges in civil engineering. It fills a key gap in functional cement-based composites by demonstrating that multi-component cements not only reduce carbon footprint—up to 39%—but also meet or exceed mechanical and durability standards required for road infrastructure. Through comprehensive testing (strength, frost resistance, water penetration, and air void analysis), the study confirms that these sustainable mixes are viable alternatives to traditional CEM I cement, offering enhanced functionality for modern, eco-conscious civil engineering applications. The study concludes that low-clinker cements (CEM II, III, V) significantly reduce concrete’s carbon footprint—up to 39%—while meeting strength and durability standards. These eco-friendly mixes are viable for sustainable road infrastructure and heavy traffic applications.

Bouchikhi et al. [20] explores the use of ground glass powder (GP) as a supplementary cementitious material to enhance the functional performance of cement-based composites, specifically for zinc (Zn) stabilization. It addresses an important gap in civil engineering materials research: the limited understanding of how SCMs like GP contribute to the immobilization of heavy metals in cement matrices. By comparing a 30% GP–70% Portland cement blend with a conventional 100% cement binder, the study demonstrates that GP significantly improves Zn immobilization over time, especially after 90 days of curing. Through microstructural, mechanical, and chemical analyses, the study reveals new fixation mechanisms involving GP, such as Zn–Si and Zn–C–S–H interactions, offering insights into designing more durable, sustainable, and pollution-resistant cement composites for infrastructure applications. The work concludes that glass powder enhances zinc stabilization in cement binders after 90 days, forming stable Zn–Ca phases. Though Zn delays hydration, GP promotes long-term immobilization, making it a viable solution for heavy metal remediation in concrete.

Rafieizonooz et al. [21] focuses on the integration of Carbon Nanotubes (CNTs) into Ultra-High-Performance Concrete (UHPC) to enhance its mechanical properties, chloride resistance, and service life. It addresses important gaps in functional cement-based composites by exploring how nanoscale reinforcement via CNTs can improve durability and structural performance, especially under aggressive environmental conditions. The study fills a research void by evaluating CNT compatibility with other advanced materials like artificial lightweight aggregates and micro hollow spheres, and by analyzing microstructure through SEM, XRD, and chloride diffusion tests. It also incorporates life service prediction modeling, offering practical insights for long-term infrastructure resilience in civil engineering. The study concludes that CNTs at 0.025–0.05% improved UHPC’s compressive strength, chloride resistance, and service life. CNT3 showed reduced performance due to poor dispersion. CNT1 and CNT2 mixes offered optimal durability and structural benefits.

Sanjuán et al. [22] investigates the carbonation resistance of ternary Portland cements incorporating silica fume and limestone, addressing key gaps in sustainable cement-based composites for civil engineering. Traditional Portland cement contributes significantly to CO_2_ emissions, and while blended cements offer environmental benefits, their carbonation resistance has been a concern. This study fills that gap by evaluating the long-term performance of ternary mixes under natural carbonation conditions. It demonstrates that with proper curing, these cements meet durability standards and enhance CO_2_ uptake, offering a viable, low-carbon alternative for structural applications. The findings support performance-based design approaches for future sustainable infrastructure. The work concludes that ternary cements with silica fume and limestone show better-than-expected carbonation resistance. With proper curing, they meet durability standards and enhance CO_2_ uptake, supporting their role in sustainable, low-carbon concrete solutions.

Suarez-Navarro et al. [23] study evaluates the radioactive content in ashes from recycled fuels used in cement production, ensuring safety in co-processing practices. It addresses an important gap in civil engineering by linking chemical composition to radiological behavior, enabling safer integration of recycled materials into functional cement-based composites. By using gamma spectrometry and principal component analysis, the study identifies correlations between radionuclide concentrations and metal oxides like Fe_2_O_3_, supporting predictive assessments. The findings confirm low radiation exposure for workers and promote sustainable, circular economy practices in cement manufacturing, advancing the development of safe, eco-friendly construction materials. The work concludes that co-processing recycled waste in cement production poses no radiological risk. Sewage sludge showed the highest natural radionuclide levels, but worker exposure remained below safety limits. Future studies should explore radionuclide leaching.

Huang et al. [24] study explores the incorporation of granite powder (GP), a byproduct of stone processing, into mass-manufactured sand concrete as a partial cement replacement. Replacing 5–10% of cement with GP enhanced 28-day compressive (up to +17.6%) and flexural strength (+20.9%), reduced autogenous shrinkage (−19.7%), and lowered hydration heat (−7.2%), mitigating thermal cracking risks in mass concrete. Microstructural analyses (SEM, XRD, MIP) revealed that GP improved pore structure and acted as nucleation sites without altering hydration products. The study addresses important gaps in functional cement-based composites by promoting sustainable reuse of industrial waste, optimizing GP dosage for performance, and providing mechanistic insights into GP’s role in enhancing durability and mechanical properties—critical for large-scale civil engineering applications. The work concludes that granite powder enhances mass-manufactured sand concrete by improving strength, reducing shrinkage and hydration heat, and refining microstructure. Optimal benefits occur at 5–10% replacement, beyond which performance may decline.

Adeleke et al. [25] study addresses an important gap in the development of functional cement-based composites for civil engineering by exploring the under-researched potential of using polylactic acid (PLA) waste plastic as a coarse aggregate in geopolymer concrete. While previous studies have focused on plastic as fine aggregates or fibers in ordinary Portland cement (OPC) concrete, this research uniquely investigates the performance of geopolymer concrete incorporating coarse recycled plastic aggregates. It evaluates both mechanical and microstructural properties, demonstrating that geopolymer concrete with up to 70% plastic replacement maintains superior strength compared to OPC mixes. The study also highlights improved interfacial bonding and reduced porosity in geopolymer matrices, offering a sustainable, high-performance alternative to traditional concrete. The work concludes that using waste plastic as coarse aggregate in geopolymer concrete, will potentially achieve notable strength and sustainability benefits. It supports greener alternatives in construction.

Abbey et al. [26] study investigates the durability and microstructural behavior of expansive subgrade soils treated with sustainable cementitious waste materials—specifically ground granulated blast furnace slag (GGBS) and brick dust waste (BDW). Through wetting–drying cycle tests, California Bearing Ratio (CBR) evaluations, and microstructural analyses (SEM and EDX), the study demonstrates that combining GGBS and BDW enhances the strength, durability, and resilience of treated subgrades under cyclic environmental stress. The formation of calcium silicate hydrate (CSH) gels and increased alumina/silica content from BDW contribute to improved interparticle bonding and reduced mass loss. The study addresses an important gap in civil engineering: the limited durability of cement-based composites under fluctuating moisture conditions. Traditional binders like cement and lime, while effective in strength enhancement, often degrade under cyclic wetting and drying. By introducing BDW—a highly pozzolanic and sustainable material—the study offers a viable alternative that not only improves mechanical performance but also aligns with environmental sustainability goals. The work concludes that Expansive subgrades treated with GGBS and BDW demonstrated enhanced durability and strength under wet–dry cycles. The pozzolanic synergy between these materials reduced mass loss and improved microstructural integrity, making them sustainable alternatives for resilient road construction in fluctuating environmental conditions.

The fourteenth text published in this Special Issue is a review by Li and Cao [27] on the recent developments on the effects of micro- and nano-limestone on the hydration process, products, and kinetics of cement. The study presents a comprehensive review of the role of limestone-based materials in cement hydration. The authors focus on three distinct forms of calcium carbonate—limestone powder (LP), calcium carbonate whiskers (CWs), and nano-calcium carbonate (NC)—and examine their effects on hydration mechanisms, product formation, and kinetics. This work is particularly relevant to the development of functional cement-based composites in civil engineering, where performance, sustainability, and durability are critical. The study begins by acknowledging the environmental challenges associated with cement production, notably its high carbon footprint and energy consumption. In response, researchers have explored supplementary cementitious materials (SCMs) to partially replace cement in concrete. Among these, limestone has emerged as a promising candidate due to its chemical compatibility, abundance, and cost-effectiveness. However, the mechanisms by which limestone influences hydration—especially at micro and nano scales—have not been fully understood or quantified. This study addresses that gap by systematically analyzing the physical and chemical effects of limestone on cement hydration. The work concludes that micro- and nano-limestone improve cement hydration via nucleation and chemical effects, while larger particles mainly dilute. Their use enhances strength, reduces CO_2_ emissions, and supports sustainable concrete development.

## 3. Conclusions

This Special Issue showcases a diverse and forward-thinking collection of research that advances the development of functional cement-based composites for civil engineering. Each contribution addresses critical gaps in sustainability, durability, and performance, offering innovative solutions that align with global efforts to reduce carbon emissions and promote eco-efficient construction. From the integration of recycled materials and nanotechnology to the optimization of mix designs and the use of alternative binders, the studies collectively highlight the transformative potential of interdisciplinary approaches in cement science [28,29].

The findings underscore the importance of leveraging locally available resources, enhancing material efficiency, and adopting performance-based design strategies. Whether through the use of geopolymer concrete, low-emission cements, or supplementary cementitious materials like glass powder and zeolite-rich tuff, the research demonstrates that sustainable construction is not only feasible but also capable of meeting stringent engineering requirements [10,29,30].

As the civil engineering sector continues to evolve in response to environmental and societal demands, the innovations presented here provide a roadmap for future research, policy development, and industrial application. By embracing these advancements, stakeholders can contribute to a resilient, low-carbon built environment that supports both infrastructure growth and ecological stewardship [31].

Future research should compare self-tapping screw and welded connections in DPSCWs under seismic conditions, optimize screw layouts for ductility, and refine design via parametric FE studies. Studies on cement composites with mineral additives like white microsilica should assess long-term durability, shrinkage, and mechanical performance. SCC design using crushed aggregates (basalt, granite) and fillers (slag, limestone flour) needs expanded testing and mix model refinement. Foamed vegetation concrete should be tested in varied climates and soils to validate field performance. Zeolite–cement ratios beyond 25% and scalability for industrial use warrant exploration. Low-emission concrete research should focus on field performance, recycled aggregates, and rapid construction mix optimization. GP–cement composites need durability studies, pollutant-specific GP content optimization, and multi-metal stabilization. CNT dispersion, durability beyond 90 days, smart sensing, and economic feasibility require attention. Carbonation models for ternary cements need refinement under diverse conditions. Radionuclide leaching from Fe_2_O_3_-rich ashes and predictive modeling should be studied. Granite powder concrete’s durability, structural applications, and synergy with other SCMs should be explored. Geopolymer concrete with waste plastic needs admixture optimization and durability studies. BDW–GGBS blends require field validation, mix optimization, life cycle analysis, and modeling. Micro/nano-limestone research should quantify nucleation area, optimize dispersion, and study hydration kinetics.
